# Nitrogen Deposition Reduces Plant Diversity and Alters Ecosystem Functioning: Field-Scale Evidence from a Nationwide Survey of UK Heathlands

**DOI:** 10.1371/journal.pone.0059031

**Published:** 2013-04-29

**Authors:** Georgina E. Southon, Christopher Field, Simon J. M. Caporn, Andrea J. Britton, Sally A. Power

**Affiliations:** 1 Division of Biology, Imperial College London, Ascot, Berkshire, United Kingdom; 2 Division of Biology and Conservation Ecology, Manchester Metropolitan University, Manchester, United Kingdom; 3 James Hutton Institute, Craigiebuckler, Aberdeen, United Kingdom; 4 Hawkesbury Institute for the Environment, University of Western Sydney, Penrith New South Wales, Australia; Jyväskylä University, Finland

## Abstract

Findings from nitrogen (N) manipulation studies have provided strong evidence of the detrimental impacts of elevated N deposition on the structure and functioning of heathland ecosystems. Few studies, however, have sought to establish whether experimentally observed responses are also apparent under natural, field conditions. This paper presents the findings of a nationwide field-scale evaluation of British heathlands, across broad geographical, climatic and pollution gradients. Fifty two heathlands were selected across an N deposition gradient of 5.9 to 32.4 kg ha^−1^ yr^−1^. The diversity and abundance of higher and lower plants and a suite of biogeochemical measures were evaluated in relation to climate and N deposition indices. Plant species richness declined with increasing temperature and N deposition, and the abundance of nitrophilous species increased with increasing N. Relationships were broadly similar between upland and lowland sites, with the biggest reductions in species number associated with increasing N inputs at the low end of the deposition range. Both oxidised and reduced forms of N were associated with species declines, although reduced N appears to be a stronger driver of species loss at the functional group level. Plant and soil biochemical indices were related to temperature, rainfall and N deposition. Litter C:N ratios and enzyme (phenol-oxidase and phosphomonoesterase) activities had the strongest relationships with site N inputs and appear to represent reliable field indicators of N deposition. This study provides strong, field-scale evidence of links between N deposition - in both oxidised and reduced forms - and widespread changes in the composition, diversity and functioning of British heathlands. The similarity of relationships between upland and lowland environments, across broad spatial and climatic gradients, highlights the ubiquity of relationships with N, and suggests that N deposition is contributing to biodiversity loss and changes in ecosystem functioning across European heathlands.

## Introduction

Human activities associated with the production of energy, fertilisers and leguminous crops have had a substantial effect on the global nitrogen (N) cycle [1]. Recent estimates suggest that anthropogenic contributions to the global reactive N pool have increased 10-fold since pre-industrial times and are predicted to double, relative to current day levels, by 2050 [2]. Deposition of N is spatially highly variable; in Europe, for example, rates currently range from ∼1 kg ha^−1^ yr^−1^ in the relatively pristine areas of northern Scandinavia to >50 kg ha^−1^ yr^−1^ in areas dominated by industry (e.g. Northern Italy) or intensive agriculture (e.g. Netherlands) [3].

The link between N deposition and changes in the structure and functioning of terrestrial ecosystems is now widely acknowledged, with N cited as one of the leading drivers of biodiversity loss at a global scale [4], [5]. Manipulation experiments have demonstrated a wide range of plant responses to N, including changes in plant phenology, physiology, biochemistry, productivity and assimilate allocation [6]–[8]. Resulting changes in competition between neighbouring species, as well as N-driven changes in soil pH and NH_4_^+^ concentrations and/or increased sensitivity to environmental stresses, are believed to be responsible for changes in plant community composition observed in many ecosystems [9]. Similarly, N-driven changes in the activity and composition of the soil microbial community [10], [11] have been linked with changes in key soil processes, such as rates of decomposition and nitrous oxide production [12]–[13], with implications for the functioning of affected ecosystems.

Although many of the responses reported in N-manipulation experiments are not specific to N and will be affected by factors such as climate and habitat management, several have been proposed as potential robust bioindicators of the effects and/or amounts of N deposition. These include foliar N accumulation, activities of enzymes involved in nutrient metabolism, soil C:N ratios and concentrations of N in soil solution and leachate [14], [15]. Relatively few studies have explicitly tested whether the potential bioindicators of (effects of) N deposition observed in manipulation experiments are apparent and/or widespread under natural, field conditions. Those studies which have done so have tended to focus on relationships between N deposition and either species richness *or* biochemical indicators, but rarely both. For example, Remke *et al.* [16] reported a decline in species richness of coastal dune systems with increasing N deposition, while in a recent analysis of UK Countryside Survey data, Maskell *et al.* [17] suggest that acidification and eutrophication associated with N deposition are responsible for biodiversity loss in UK grasslands and heathlands. Surveys focusing on biochemical indicators of N deposition identified in manipulation experiments (e.g. tissue N concentrations, soil nutrient availability, litter chemistry and/or enzyme activities) have provided evidence of field-scale changes in ecosystem functioning, particularly in relation to nutrient cycling, along gradients of ambient N deposition [18]–[20]. A small number of studies have demonstrated links between diversity loss, changes in ecosystem function and N deposition, including a regional survey of moorlands [18], and both a national [21] and pan-European [22] survey of acidic grasslands. To date, however, there has been no wide-scale evaluation of the relationships between community-level (compositional) bioindicators, biogeochemical (functional) indicators and N deposition in heathland ecosystems, despite the fact these have been shown to respond strongly to experimental N manipulation [23]–[26].

Atmospheric N occurs in both oxidised (NO_y_) and reduced forms (NH_x_); anthropogenic emissions are associated principally with the combustion of fossil fuels (oxidised N) and intensive agricultural practices (reduced N) processes [27]. Whilst ecosystem responses to increasing total N deposition have been extensively researched, far less is known about the sensitivity of soils and vegetation to the different forms of N [27]. Findings from the relatively few studies that have investigated the role of different forms of N have reported differences in the strengths of relationships between oxidised and reduced forms in terms of plant tissue chemistry [28] species richness and community composition [29], [30].

Upland and lowland heathlands are typically of low nutrient status and dominated by dwarf shrubs. However, their contrasting altitudes (lowlands typically <300m, uplands >300m [31]) result in characteristically different levels of soil organic matter, rates of nutrient cycling, plant species assemblages and management regimes [32] which might be expected to result in differing responses to elevated N inputs. Upland and lowland habitats also experience different climates (typically wetter and cooler in the former compared to the latter), which have the potential to modify their sensitivity to atmospheric N inputs [33]. Indeed, although N manipulation experiments have demonstrated similar qualitative responses to N in upland and lowland heathlands (e.g. increased *Calluna* productivity and reduced abundance of lower plants [24], [34], [35]), differences in terms of soil biogeochemical responses have been found [23], [26]. The ubiquity of heathland responses to N across a range of altitudinal and climatic gradients has not yet been tested. This study, therefore, presents the first comprehensive, nationwide survey of biological and biogeochemical indicators of N deposition across lowland and upland heathlands. It explicitly aims to evaluate relationships between N deposition and 1) plant community composition and species richness and 2) ecosystem functioning, as measured by soil nutrient stocks, availabilities and enzyme activities. Based on results from earlier manipulation experiments and surveys, we hypothesise that similar relationships with N deposition will be evident in upland and lowland heaths, and that reduced forms of N are having a greater impact than oxidised N on the diversity and functioning of UK heathlands.

## Methods

### Site selection and field surveying

**Figure 1:**
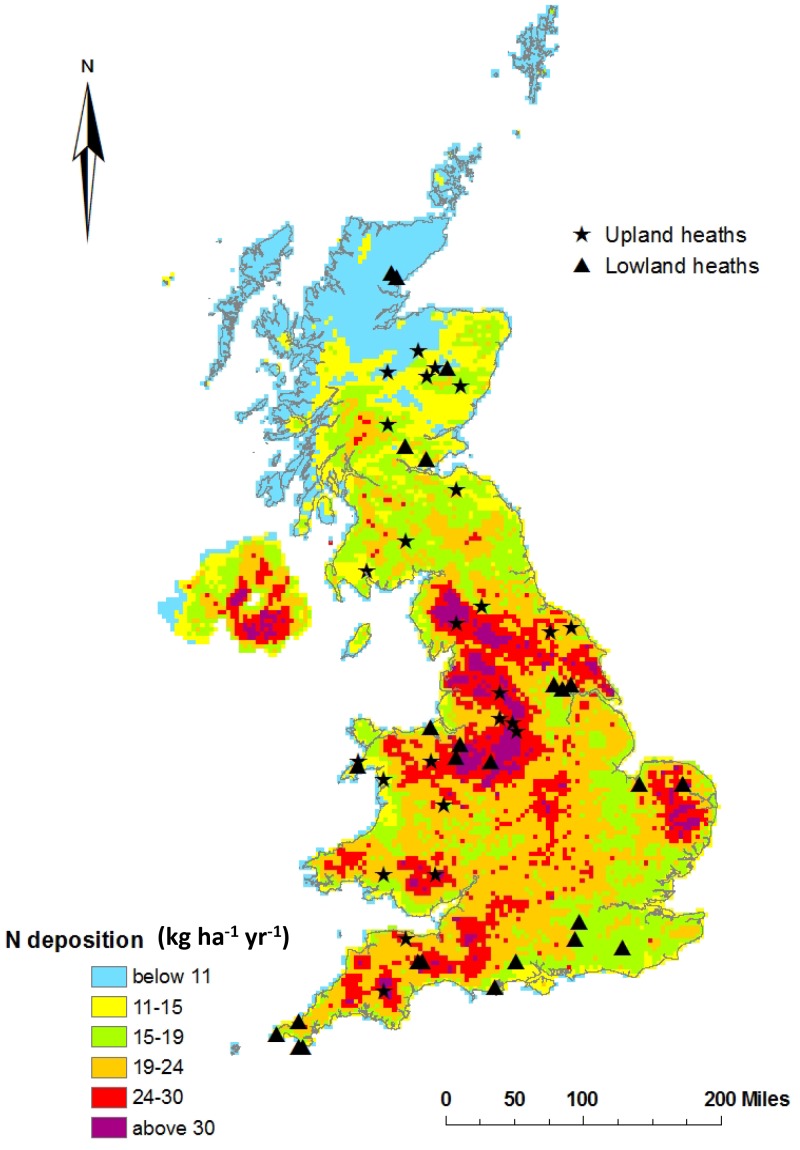
Site survey locations in relation to total N deposition (2006). (http://pollutantdeposition.defra.gov.uk/pollutant-maps).

Fifty two *Calluna vulgaris* dominated heathland sites (representing building to mature phase stands, *sensu* Gimingham, [32]) were selected across a gradient of modelled N deposition (data provided by Ron Smith and Jane Hall, Centre for Ecology & Hydrology, Edinburgh, UK) spanning 5.9 kg N ha^−1^ yr^−1^ to 32.4 kg N ha^−1^ yr^−1^ (Figure 1). In addition, sites were chosen to be representative of the geographical, climatic and altitudinal (25 upland and 27 lowland heaths) variation encountered in heathland ecosystems across the United Kingdom.

Sites were visited during the summer of 2009 and grid reference and altitudinal details were recorded at each site. Vascular and non-vascular plant species were recorded and relative abundance was estimated by percentage cover in five randomly placed quadrats (2 m×2 m) at each site. Canopy height was measured to the nearest mm at the centre of each of the four 1 m^2^ quadrants comprising the 2 m×2 m quadrat, and averaged. 5–10 g of *Calluna* shoots were collected into paper bags and air dried for later chemical analyses. Three soil cores (15 cm depth ×4 cm diameter) were extracted from the centre of the fifth quadrat for chemical analyses; these were stored in an ice-filled cool-box before transfer to controlled temperature rooms upon return to the laboratory, typically within 1–3 days. Five samples of fresh *Calluna* litter (5–10g) were also collected from each quadrat for enzyme assays. The litter was air dried and stored in paper bags.

### Chemical analyses

To determine extractable soil ammonium and nitrate concentrations, 40 g of fresh soil was mixed with 150 ml of 1M KCl and shaken for one hour on a rotary shaker before filtering. The filtrate was measured for inorganic N (NH_4_^+^ and NO_3_^−^) using a SKALAR san flow^++^ segmented flow analyser (Skalar UK (Ltd), York, UK). Litter phosphomonoesterase (PME) activity was assayed using the artificial substrate p-nitrophenyl phosphate (p-NPP), in accordance with the method outlined by Johnson *et al.* [36]. Samples were centrifuged for 5 minutes at 3000 rpm and the resulting liquid was analysed colourimetrically (using a Perkin Elmer Lambda 3 UV spectrophotometer, Perkin Elmer, USA) at a wavelength of 460 nm. Paired non- reactive controls were prepared for each of the samples by adding the PNP and toluene solution immediately after the stopping chemicals to prevent chemical reaction. The readings were compared with pigment densities from a calibration curve obtained using six standards of p-nitrophenol prepared within the absorbance range of the samples.

Total N and carbon (C) concentrations were determined using a FlashEA 1112 CN analyser (Thermo Scientific, UK). *Calluna* shoot and litter samples were dried at 80°C for 24 hours before being ground into a fine powder with a ball mill for two minutes. 15–20 µg of milled material was weighed into tin capsules and combusted. Standard samples were prepared using certified reference materials. Soil pH _(H2O)_ was measured by mixing 10g field moist soil with 25ml deionised water, then allowing the mixture to stand for 30 minutes; pH was then recorded using a CORNING 220 pH meter (Corning Inc., USA).

Litter phenol oxidase activity was measured by vortex-mixing 1 g of ground *Calluna* litter with 9 ml deionised water. 3 ml of this solution was then added to 4.5 ml water and 7.5 ml of 1 M dihydroxyphenylalanine (DOPA) and then shaken for 9 minutes. After shaking, the mixture was centrifuged at 6000 rpm for 5 minutes. A sub-sample was then taken, filtered (0.2 µm) and absorbance measured at 460 nm using a Perkin Elmer Lambda 3 UV spectrophotometer, Perkin Elmer, USA. Soil moisture content was determined gravimetrically by weighing a 10g sample of fresh soil, drying this at 105°C for 24 hours, then re-weighing.

### Data analyses

Relationships between response and explanatory variables (N deposition, rainfall and temperature) were analysed using the R statistical package, version 2.12.2 (R development core team, 2011). The climate data used were based upon UK 5 km^2^ gridded data sets from the UK Meteorological Office. Variables representing total annual precipitation and temperature were used, the latter represented by data on growing degree days (GDD, sum of degree days above 5°C in a given year). Both precipitation and GDD were averaged over the period 1997–2006. Since total deposition is the product of oxidised and reduced N deposition these three explanatory variables could not be included in the same statistical model. In order to separate the combined effects of total N deposition from the individual influences of oxidised and reduced N forms, two separate models were, therefore, run for each response variable. Model 1  =  total N deposition, rainfall and temperature; Model 2  =  reduced N, oxidised N, rainfall and temperature. Percentage cover data for vegetation were arc-sine transformed prior to analysis. Log or square root transformations were used to transform other non-normally distributed data prior to modelling. Relationships were tested using multiple regression analysis or, for count data (e.g. species richness), generalised linear models (GLM) with poisson errors. Relationships with species richness were investigated firstly for all species recorded at a site and subsequently for the different plant functional groups present (lichens, mosses, grasses and forbs). Where curvature in the data was evident, models were fitted with a quadratic function. The most complicated models were fitted (to include interactions between explanatory variables, plus quadratic terms to test for non-linearity of responses) and then simplified using likelihood ratio deletion tests until the minimum adequate model was attained. Evidence of overdispersal was corrected by re-fitting the model with quasi-empirical structures, followed by F tests as outlined by Crawley [37]. Three analyses were performed for each parameter to encompass (a) all sites, (b) upland sites only and (c) lowland sites only.

## Results

### Higher plant responses

**Figure 2:**
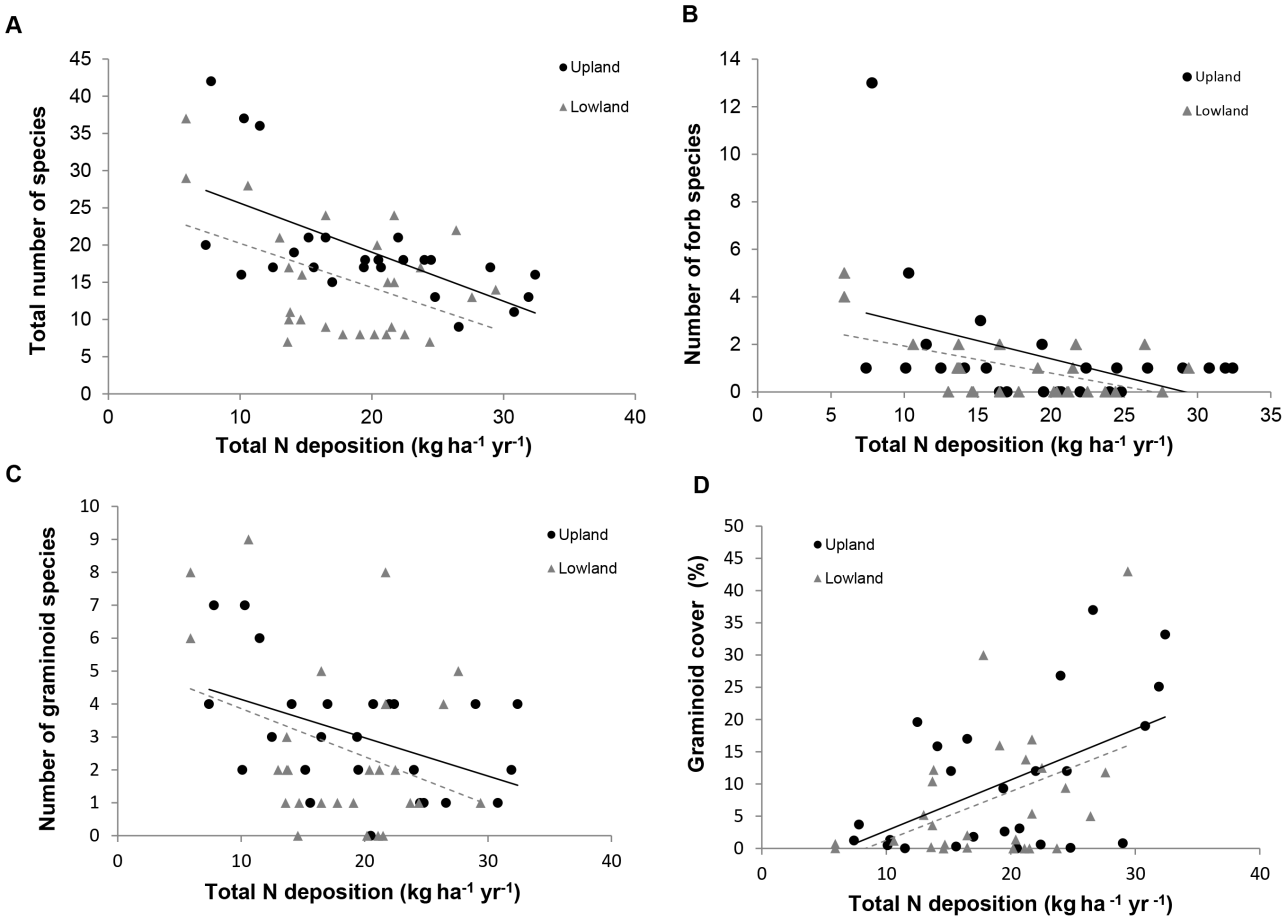
Relationships between N deposition and plant species richness and abundance. A decline of species richness across all plant groups correlates with increasing N deposition (A) and where N deposition and temperatures are higher (B). Whilst graminoid species richness declines in relation to increased N deposition inputs (C), graminoid abundance increases (D), indicating the promotion of fewer species at the higher end of the N gradient.

A significant decline in overall species richness was observed across all sites in relation to increasing levels of total N deposition (F_1,50_ = 27.5, P<0.001, Figure 2*a*). The biggest losses were seen at the lower end of the N deposition gradient, with an average of 13 species lost per site between 5–10 kg N ha^−1^ yr^−1^, compared to an average of only 3 species lost as N increased from 10 to 20 kg N ha^−1^ yr^−1^. Beyond this, declines in species richness plateaued, indicating a reduction in sensitivity as N loading increased. Temperature was also a significant factor across all sites, both on its own and in interaction with N deposition (F_1,50_ = 4.4, P<0.05, Table 1: model 1). Species richness was seen to decline by 50 % across all sites around a temperature threshold of 1000 growing degree days (GDD) and total N inputs of 15 kg N ha^−1^ yr^−1^, and declined by a further 50 % when GDD exceeded 2000; this relationship was significant in the lowland-only dataset and as a not quite significant trend for the uplands.

**Table 1:** Summary of optimal models for plant species richness in relation to N deposition and climate variables.

	Model 1 (Total N deposition + environmental variables)									Model 2 (oxidized/reduced N + environmental variables)								
Response variable	All sites	d.f.	P	Uplands	d.f.	P	Lowlands	d.f.	P	All sites	d.f.	P	Uplands	d.f.	P	Lowlands	d.f.	P
Total species	(−) N	51	***	(−) N	23	***	(−) N	26	**	(−) NO_y_	51	**	(−) NO_y_	24	***	(−) NO_y_	26	**
	(−) gdd		***				(−) gdd		**	(−) gdd		***	(−) NH_x_		*	(−) gdd		**
	N x gdd		*							(−) NH_x_		***						
																		
Total lichen species	(−) N	51	**	(−) N	24	**	-	-	-	(−) NH_x_	51	***	(−) NO_y_	24	**	(−) NH_x_	24	*
	N x gdd		*	N x gdd		**				(−) NO_y_		**	(−) NH_x_		*			
										(−) gdd		***	NH_x_ x gdd		**			
													NH_x_ x rain		*			
																		
Total bryophyte species	(−) gdd	51	***	-	-	-	(-) gdd	26	**	(-) gdd	51	***	-	-	-	(−) NOy	26	**
																(−) gdd		*
																(−) rain		*
																		
Total graminoid species	(−) N	51	**	(−) N	24	*	(−) N	26	*	(−) NO_y_	51	*	(−) NH_x_	24	*	(−) NO_y_	26	*
	(−) gdd		**				(−) gdd		**	(−) NH_x_		*				(−) gdd		***
										(−) gdd		**				NH_x_ x gdd		*
																		
Total forb species	(−) N	51	***	(−) N	24	**	(−) N	26	**	(−) NO_y_	51	***	(−) NO_y_	24	***	(−) NO_y_	26	***
				N x rain		*				(−) NH_x_		*	(−) NH_x_		*	NO_y_ x rain		*
										(−) gdd		*				NH_x_ x rain		*
																		

(+/−)  =  direction of response; x  =  interaction; P values  =  * <0.05, **<0.001, ***<0.001; gdd  =  growing degree days.

The number of forb species also decreased with increasing total N deposition (F_1,50_ = 21.2, P<0.001, Table 1: model 1; Figure 2b), with the steepest rate of decline seen with modest increases of N at the lower end of the deposition gradient (4 species lost on average per site between 5–10 kg N ha^−1^ yr^−1^ with negligible losses after this). Detailed analysis of the role of N forms indicates that, for forbs across all sites, stronger relationships were found with oxidised N (F_1,50_ = 16.0, P<0.001, Table 1: model 2). Significant interactions between N deposition and rainfall indicate that forb diversity decline is exacerbated in wetter areas receiving higher levels of N deposition. In the uplands, the relationship was significant with total N deposition (F_1,19_ = 6.9, P<0.05, Table 1: model 1), whilst in the lowlands, NO_y_ and NH_x_ both had significant interactions with rain (F_1,20_ = 4.6, P<0.05, F_1,19_ = 6.2, P<0.05 respectively) Table 1: model 2).

Graminoid species richness declined significantly across all sites with increasing total N inputs (F_1,50_ = 9.3, P<0.01, Figure 2*c*), as well as with increasing temperature (F_1,50_ = 10.9, P<0.01). Losses associated with increasing N were highest at the lower end of the deposition range (an average of 4 species lost per site between 5–10 kg ha^−1^ yr^−1^) with a slower rate of loss as inputs increased further. Additionally, each incremental temperature increase of 500 growing degree days corresponded to an average loss of 1 species per site. Across all sites, both forms of N (oxidised and reduced) were associated with graminoid species diversity decline. However, when upland and lowland datasets were considered independently, stronger relationships with NO_y_ were found in the lowland sites, and with NH_x_ in the upland sites. Graminoid abundance, by contrast, increased with increasing N (F_1,50_ = 15.8, P<0.001), indicating a shift towards more grass dominated systems with greater N deposition (Figure 2*d*); reduced N was the most significant driver of this response (F_1,50_ = 19.5, P<0.001, Table 2: model 2). Increases in grass abundance were, however, found to be chiefly characterised by a few species, specifically *Molinia caerulea* and *Deschampsia flexuosa*, the latter being significantly more abundant at sites receiving greater reduced N inputs (F_2,49_ = 9.2, P<0.01, Table 2: model 1).

**Table 2:** Summary of optimal models for vegetation responses in relation to N deposition and climate variables.

	Model 1 (total N deposition + environmental variables)									Model 2 (oxidized/reduced N + environmental variables)								
Response variable	All sites	d.f.	P	Uplands	d.f.	P	Lowlands	d.f.	P	All sites	d.f.	P	Uplands	d.f.	P	Lowlands	d.f.	P
Graminoid cover (%)	(+) N	50	***	(+) N	23	**	(+) N	22	**	(+) NH_x_	50	***	(+) NH_x_	23	**	(+) NH_x_	25	**
							gdd x rain		**									
																		
Bryophyte cover (%)	(−) gdd	50	***	(−) gdd	23	*	(−) gdd	25	**	(−) gdd	50	***	(−) gdd	25	*	(−) gdd	25	**
																		
Lichen cover (%)	(−) N	47	**	(−) N	20	*	(−) N	25	*	(−) NH_x_	47	**	(−) NO_y_	19	*	(−) NH_x_	25	**
	(+) rain		*	(+) rain		**				rain x ht		***	(+) rain		***			
	rain x ht		**	rain x ht		***							NO_y_ x ht		*			
													rain x ht		***			
																		
Canopy height (cm)	(+) N	49	***	(+) N	22	***	(+) gdd	25	*	(+) NH_x_	49	***	(+) NH_x_	22	***	(+) gdd	25	*
	(+) gdd		***	(+) gdd		***				(+) gdd		**	(+) gdd		***			
																		
*Pleurozium* (%)	(−) N	48	***	(+) rain	22	**	(−) N	24	***	(−) NO_y_	47	**	(+) rain	22	**	(−) NH_x_	24	**
	(+) rain		***	N x rain		***	(−) gdd		**	(−) NH_x_		***	NH_x_ x rain		***	(−) gdd		**
	N x rain		***							(+) rain		***						
										NH_x_ x rain		***						
																		
*Brachythecium* (%)	(+) N	50	*	-	-	-	-	-	-	(+) NH_x_	50	*	-	-	-	-	-	-
																		
*Hylocomnium* (%)	(−) N	49	**	(−) gdd	23	*	(−) gdd	25	*	(−) NH_x_	48	**	(−) gdd	23	*	(−) NO_y_	23	*
	(−) gdd		**							(−) gdd		**				(−) gdd		*
																		
*Deschampsia* (%)	(+) N	50	***	(+) N	23	**	-	-	-	(+) NH_x_	49	***	-	-	-	(+) NH_x_	25	*
										(−) gdd		*						
																		

(+/−)  =  direction of response; x  =  interaction; P values  =  * <0.05, **<0.001, ***<0.001; gdd  =  growing degree days; ht  =  *Calluna* canopy height.

Across both models and all sites, significant positive relationships were observed between *Calluna* canopy height and temperature (F_2,49_ = 12.6, P<0.001, Table 1: model 1). Whilst height increased in a linear fashion in relation to total N deposition across all sites (F_2,49_ = 15.3, P<0.001, Table 2: model 1), this relationship was largely driven by NHy in the uplands (F_2,22_ = 24.8, P<0.001, Table 2: model 2).

### Lower plant responses

**Figure 3:**
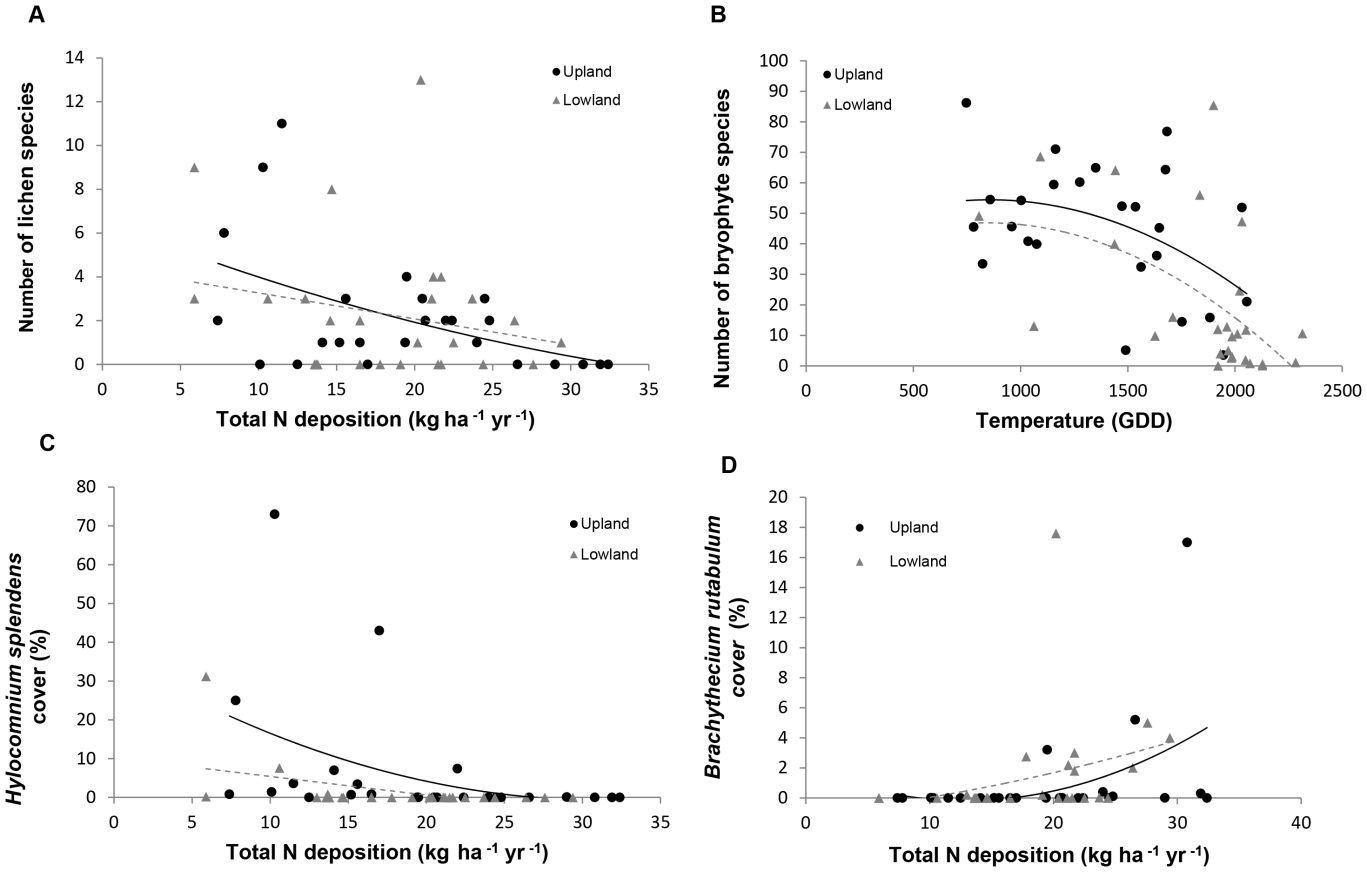
Relationships between N deposition and lower plant species richness and abundance. Declines in lichen species richness are related to increasing N deposition (A) whereas bryophyte richness per se is principally influenced by temperature (B). At a species level, however, significant species-specific relationships between N deposition and bryophytes were evident (C, D).

Significant negative relationships between lichen species richness and total N deposition were found across the combined (all sites) dataset (F_1,50_ = 7.8, P<0.01, Table 1: model 1, Figure 3*a*), driven largely by upland sites. An average of 5 lichen species per site was recorded in areas receiving less than 10 kg N ha^−1^ yr^−1^. This declined to an average of 1.4 species at input rates of 20 kg ha^−1^ yr^−1^, indicating that even modest rates of N loading are associated with a considerable loss of lichen species. Significant interactions indicate that the negative effects of N deposition on lichen richness were greater at sites with high temperature (F_1,48_ = 4.8, P<0.05, Table 1: model 1), particularly in the uplands (F_1,20_ = 8.9, P<0.01, Table 1: model 1).

Considered independently, patterns in lichen abundance at upland and lowland sites were best explained by the different forms of N. The relationship with oxidised N was statistically stronger in upland sites (F_5,19_ = 4.5, P<0.05, Table 2: model 2), whilst the relationship was stronger with reduced N in the lowlands (F_1,25_ = 8.9, P<0.01, Table 2: model 2). Climatic conditions were also important (particularly in the wetter, upland areas), with lichen abundance generally greater in areas receiving more rainfall (F_4,47_ = 4.4, P<0.05, Table 2: model 1). Lichen cover was also generally lower in wetter areas with taller *Calluna* canopies (F_4,47_ = 9.1, P<0.001, Table 2: model 1).

Temperature was the main driver influencing bryophyte species richness and abundance across all sites (F_1,50_ = 21.3, P<0.001, Table 1: model 1, Figure 3*b*), with less species recorded at the warmer end of the temperature gradient (<1000 GDD  =  10 species (on average) per site; > 2000 GDD  =  5 species (on average) per site). This overall relationship was driven by the strong decline at lowland sites experiencing the warmest conditions, with a non-significant relationship in the uplands dataset. Whilst a general downward trend in bryophyte diversity was apparent in relation to N deposition across all sites, the only significant N signal was found in the lowland sites in the form of oxidised N (F_1,26_ = 11.2, P<0.01, Table 1: model 2). Stronger relationships with N were, however, found at the species level: the characteristic heathland mosses *Hylocomium splendens* (Figure 3*c*) and *Pleurozium schreberii* were seen to severely decline with increasing N (Table 2) whilst, conversely, the nitrophilous species [38] *Brachythecium rutabulum* (Figure 3*d*) was more abundant at sites receiving higher N inputs (F_1,50_ = 6.1, <0.05).

### *Calluna* tissue chemistry

**Figure 4:**
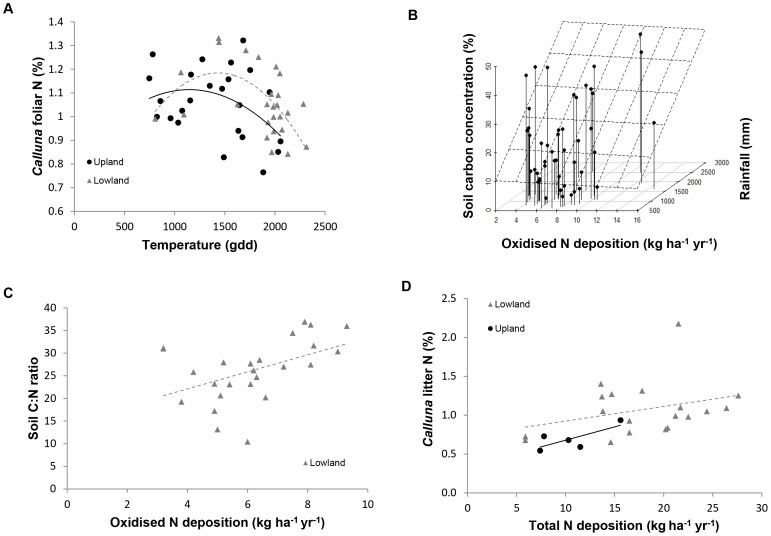
The influence of N deposition and climate on heathland plant and soil chemistry. *Calluna* foliar N concentrations were principally related to changes in temperature (A). However, positive relationships were seen between *Calluna* litter N concentrations and total N deposition (B). Soil carbon concentrations were higher in areas receiving greater oxidised N inputs and rainfall (C), and soil C:N ratios were also positively related to levels of oxidised N deposition (D).

The main factor affecting *Calluna* tissue N concentrations across all sites was temperature (Figure 4*a*). A significant quadratic relationship was found between (increasing) temperature and (higher) tissue N concentrations up to a threshold equivalent to 1500 GDD (F_1,48_ = 5.4, P<0.05, Table 3: Model 1). When upland and lowland datasets were considered separately, this relationship was only significant for the lowlands (F_2,23_ = 5.5, P<0.05).

**Table 3:** Summary of optimal models for plant and soil biogeochemical responses in relation to N deposition and climatic variables**.**

	Model 1 (Total N deposition + environmental variables)									Model 2 (oxidized/reduced N + environmental variables)								
Response variable	All sites	d.f.	P	Uplands	d.f.	P	Lowlands	d.f.	P	All sites	d.f.	P	Uplands	d.f.	P	Lowlands	d.f.	P
Soil pH	(−) N	49	**	(−) N	20	***	-	-	-	(−) NO_y_	47	***	(−) NO_y_	23	***	-	-	-
	(+) gdd		**	gdd x rain		*				(+) gdd		*						
				N x rain		*				NH_x_ x rain		*						
Extractable NO_3_	gdd x moist	38	**	(+) moist	12	***	(+) moist	24	**	(+) moist	36	***	(+) NH_x_	10	*	(+) moist		*
										gdd x moist		*	(+) moist		*			
Extractable NH_x_	(+) gdd	44	**	(+) N	11	**	-	-	-	(+) gdd	44	**	(+) NO_y_	10	**	-	-	-
				(+) moist		*							(+) gdd		*			
Total soil N	(+) rain	45	***	N x rain	19	**	-	-	-	NO_y_ x rain	44	***	(+) rain	18	*	(−) NO_y_	23	*
Total soil C	(+) rain	45	***	(−) rain	17	*	-	-	-	NO_y_ x rain	44	***	NO_y_ x rain	19	**	-	-	-
				N x rain		*												
				gdd x rain		*												
Soil C:N ratio	(−) rain	43	*	-	-	-	-	-	-	(−) rain	43	*	-	-	-	(+) NO_y_	22	**
Litter Ph-Oxidase	(−) gdd	44	*	-	-	-	gdd x rain	19	**	(−) gdd	44	*	(+) NH_x_	19	**	NH_x_ x gdd	19	**
	gdd x rain		*							gdd x rain		*	NO_y_ x rain		**			
Litter PME	(−) rain	47	***	(+) N	21	*	-	-	-	(+) NH_x_	46	*	(−) rain	21	*	(+) NO_y_	23	*
	rain x N		**	(−) rain		*				(−) rain		***						
										NH_x_ x rain		**						
*Calluna* litter N	(+) N	23	*	(+) N	2	*	-	-	-	-	-	-	(+) NH_x_	2	*	-	-	-
				(−) gdd		*							(−) gdd		*			
*Calluna* litter C:N	(−) N	23	**	-	-	-	-	-	-	(−) NH_x_	23	**	-	-	-	(+) NH_x_	18	*
*Calluna* foliar N	(−) gdd	48	*	-	-	-	(+) gdd	20	*	(+) gdd	48	*	-	-	-	NH_x_ x gdd	22	*
							N x rain		*									

(+/−)  =  direction of response; x  =  interaction; P values  =  * <0.05, **<0.001, ***<0.001; gdd  =  growing degree days; moist  =  soil moisture; PME  =  phosphomonoesterase.

### Soil pH

Soil pH ranged from 3.5 to 5.4, decreasing significantly in relation to total N deposition (F_2,49_ = 12.0, P<0.01, Table 3: model 1) and increasing at sites with higher temperatures (F_2,49_ = 7.5, P<0.01, Table 3: model 1) across all sites. The relationship with N appears to be driven by oxidised N (F_4,47_ = 13.7, P<0.001, Table 3: model 2), particularly in the upland sites.

### Extractable soil nutrients

A positive relationship between reduced N deposition and extractable soil nitrate (NO_3_^−^) concentrations was found for upland sites (F_3,10_ = 9.6, P<0.05, Table 3: model 2). Across the combined, all sites dataset, however, interactions between temperature and soil moisture (F_2,38_ = 6.9, P<0.01) suggest that extractable NO_3_^−^ concentrations are greater in areas with clement climatic conditions (i.e. warm and wet).

Extractable soil ammonium (NH_4_^+^) concentrations also increased with increasing total N in the upland sites (F_2,11_ = 11.9, P<0.01, Table 3: model 1), although this pattern was not significant in the lowlands. As with NO_3_^−^, significant relationships with climate indicate that extractable NH_4_^+^ concentrations are higher in warmer areas (F_4,9_ = 8.6, P<0.05, Table 3: model 2); this relationship appears to be driven by the uplands dataset, where greater soil NH_4_^+^ availability is also associated with higher rainfall (F_4,9_ = 18.1, P<0.01, Table 3: model 2).

### Total soil nitrogen and carbon concentrations

Significant interactions between total N deposition and rainfall were found for total soil N (F_2,19_ = 10.7, P<0.001, Table 3: model 1) and C (F_4,17_ = 6.5, P<0.05, Table 3: model 1) in upland sites, with the highest concentrations occurring at wetter sites receiving high N inputs. Model 2 analyses show that oxidised N is driving this relationship (N: F_2,44_ = 23.9, P<0.001; C: F_2,44_ = 9.3, P<0.001, Table 3, Figure 4*b*). At lowland sites, a negative relationship was seen between oxidised N and total soil N concentrations, although this was only significant at the 5% level (F_1,23_ = 6.3, P<0.05, Table 3: model 2). Climate is clearly also an important driver of soil C and N, with significantly higher concentrations at sites receiving more rainfall (N, F_1,45_ = 15.1, P<0.001; C: F_1,45_ = 13.3, P<0.001, Table 3: model 1). Indeed, at upland sites, an interaction between temperature and precipitation indicates that soil N and C concentrations are highest where rainfall and temperature are both high (N: F_3,18_ = 9.7, P<0.01, model 2; C: F_4,17_ = 5.8, P<0.05, model 1). At lowland sites, soil C:N ratios were highest at sites receiving greater oxidised N inputs (F_2,22_ = 9.0, P<0.01, Table 3: model 2, Figure 4*c*).

### Litter chemistry, enzyme activity and nutrient turnover

Across all sites, litter N concentrations were significantly higher in areas of higher N deposition (F_1,23_ = 5.3, P<0.05, Table 3: model 1, Figure 4*d*). This relationship was driven by data from upland sites, with the strongest patterns seen in relation to reduced N (F_2,2_ = ,34.0, P<0.05, Table 3: model 2). Litter C:N ratios were lower at sites with the highest N deposition (F_1,23_ = 8.6, P<0.01, Table 3: model 1); this relationship was strongest in the lowlands for reduced N (F_1,18_ = 5.5, P<0.05, Table 3: model 2), with a similar (non-significant) trend apparent for the uplands.

**Figure 5:**
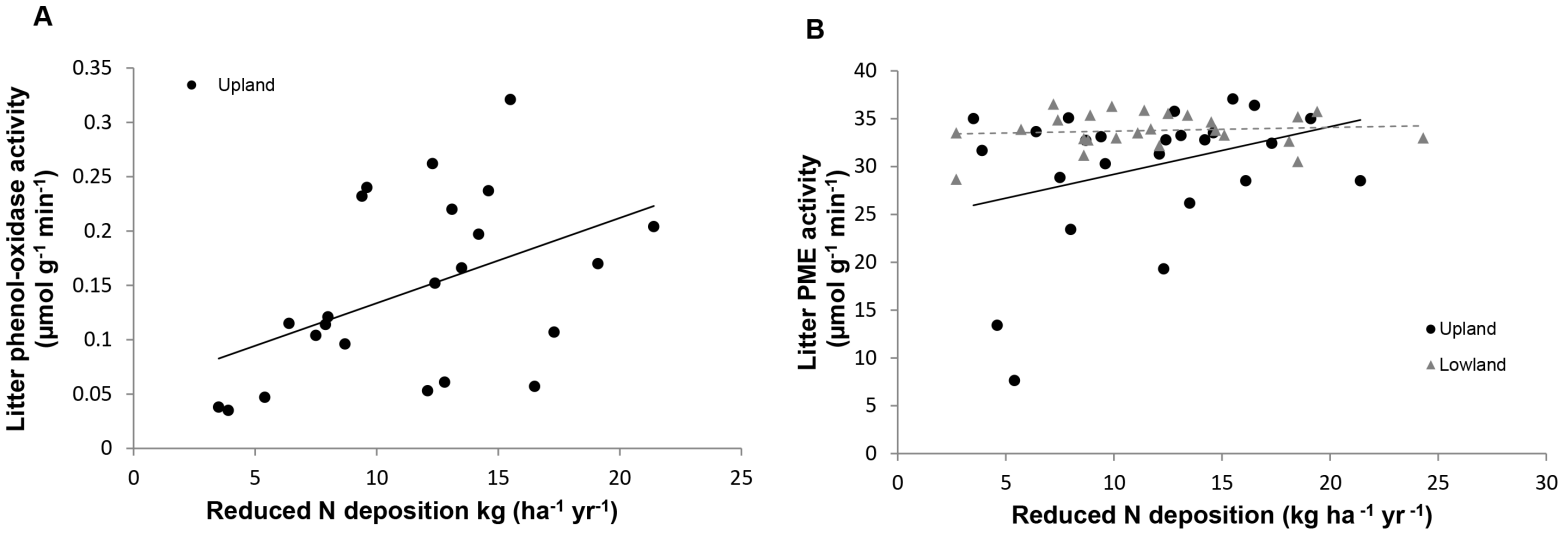
Relationships between N deposition and litter enzyme activity. Increasing reduced N deposition was positively correlated with increased litter phenol-oxidase activity in upland sites (A) and litter phosphomonoesterase (PME) activity across all sites (B).

Overall, litter phenol oxidase activity was more strongly related to climate than to levels of N deposition. Across the full (all sites) data set, enzyme activities were lower at warmer sites (F_2,44_ = 6.1, P<0.05, Table 3: model 1); a significant interaction was also apparent between temperature and rainfall, with the lowest activities recorded from areas where higher temperatures combined with greater quantities of rainfall (F_2,44_ = 6.7, P<0.05, Table 3: model 1). In upland areas, phenol-oxidase activity was, however, higher at sites receiving greater inputs of reduced N (F_3,19_ = 8.3, P<0.01, Table 3: model 2, Figure 5*a*), as well as those with high levels of both rainfall and oxidised N deposition (F_3,19_ = 12.2, P<0.01, Table 3: model 2).

As for phenol-oxidase, litter PME activity across all sites was significantly related to climate, with activity decreasing as precipitation levels increased (F_2,47_ = 15.0, P<0.001, Table 3: model 1). A positive relationship between total N deposition and litter PME activity was, however, apparent for upland sites (F_2,21_ = 4.3, P<0.05, Table 3: model 1). Analysis of N forms revealed that this was driven by reduced N and that this relationship was also significant for the all sites dataset (F_3,46_ = 4.3, P<0.05, model 2, Figure 5*b*).

## Discussion

### Above-ground responses

A decline in plant species diversity in relation to increasing atmospheric N deposition has been observed in a variety of ecosystems ranging from acid and calcareous grasslands [39], [40] to boreal and temperate forests [41], [42] and even arctic tundra [43]. Measurement of plant species richness can provide a reliable indication of habitat quality [44], [45] and is considered to be a fundamental tool in assessing community and wider-scale diversity [46]. Where species losses are countered by increases in a few, dominant and/or invasive species, the risk to habitats are homogenisation with declines in genetic-, ecosystem- and landscape-scale diversity. The findings from this survey provide clear evidence that elevated N deposition significantly reduces the floristic diversity of heathland ecosystems, either solely or in combination with climatic drivers – particularly increasing temperature – a major driver of global biodiversity loss in both terrestrial and non-terrestrial ecosystems [4], [47]. The nature of the relationship between total N deposition and species richness suggests similar patterns in both upland and lowland systems, namely that the greatest proportional reduction of species richness in response to increasing N inputs is seen at sites currently experiencing the lowest deposition loads. It appears that both oxidised and reduced forms of N are contributing to heathland biodiversity loss, although, at the functional group level there were more significant relationships between species richness and reduced N, compared to oxidised N, as hypothesised. This is in line with findings from similar surveys elsewhere for grassland [48) and heathland [19], providing further support for an additional impact associated with the acidifying properties of reduced N and/or direct toxicity of ammonium ions, over and above that of N-enrichment *per se*. Given the broad cover of spatial, climatic and pollution indices, the magnitude of diversity loss that has been observed amongst forb, graminoid, bryophyte and lichen functional groups in this study not only validates experimental findings from many N manipulation studies [39], [49] but, more profoundly, provides compelling evidence that N deposition is driving biodiversity loss amongst UK heathlands at a national scale.

In N-limited environments, increased N availability frequently leads to increased plant growth and/or expansion of nitrophilous species, potentially leading to competitive exclusion of slower growing habitat-specialist species adapted to low levels of N availability [40]. Effects of N on soil pH, particularly in its reduced form [50], can also result in the decline of sensitive plant species, particularly those with narrow pH niches or low tolerance of high NH_4_^+^ ion concentrations [51], [52]. In the current study, *Calluna* plants were taller and soil pH lower in areas receiving high N inputs, suggesting that reduced understorey light availability and/or unfavourable soil pH may be mechanisms contributing to biodiversity loss in British heathlands. Whilst bryophyte cover was not related *per se* to N deposition, shifts within bryophyte communities, notable at the species level, were significantly related to N inputs. For example, the characteristic heathland moss species *Hylocomnium splendens* and *Pleurozium schreberii* were seen to decline as N deposition increased, whilst *Brachythecium rutabulum*, a nitrophilous species more commonly associated with base rich, eutrophicated habitats [53] was more abundant at sites with higher N deposition. Similar species shifts have been seen in boreal forest systems [41], [54] with *H. splendens* declining, and being replaced by *B. rutabulum*, at relatively modest input rates of 10 kg N ha^−1^ yr^−1^.

Although graminoid abundance increased significantly with increasing N deposition, a concurrent decrease in graminoid diversity, coupled with sharp declines in the abundance of typical heathland grass species (e.g. *Festuca ovina*, *Danthonia decumbens*) indicates that grass invasion at higher N deposition loads is dominated by only a small number of species (specifically *Deschampsia flexuousa* or *Molinia caerulea*). These findings support earlier observations of *D. flexuousa* and *M. caerulea* increases in high N deposition areas in the Netherlands [55] and an associated conversion of 35 % of Dutch heathlands to grasslands in the 1980s [56]. These species have also been found to increase in Danish bog systems exposed to N levels of 10–15 kg ha^−1^ yr^−1^, to the detriment of characteristic ombrotrophic vegetation [57].

N deposition is associated with negative impacts on lichen communities in many ecosystems [58]–[60], a phenomenon often linked to increased competition for light as a result of N-driven stimulation in higher plant growth [9], [61]. However, direct toxicity of NO_2_^−^, NO_3_^−^ and NH_4_^+^ is also likely to be a contributing factor in the decline of non-vascular plants [16], [62], [63]. Whilst lichen abundance was negatively related to both reduced and oxidised forms of N in this study, declines in both the cover and species richness of lichens were statistically stronger with reduced N. As with most higher plants, lichens can effectively assimilate NH_4_^+^ at modest levels [64] rapidly converting it into amino acids to mitigate its cytotoxicity. However, for acidophytic species (such as those found in heathlands) this assimilation capacity is limited and toxic levels of unassimilated NH_4_^+^ can accumulate in the lichen thallus [65]. It seems likely, therefore, that both competitive exclusion and potentially also direct toxicity effects are contributing to the decline in abundance and diversity of lichens observed with increasing N deposition in the current study.

### Below-ground responses

At all sites in this study, litter N concentrations increased, and C:N ratios decreased, with increasing N inputs suggesting greater availability of organic N for soil microorganisms and thus faster rates of nutrient cycling. Microbial enzyme activity provides a useful proxy for decomposition rates and has been frequently used to monitor the functional responses of microbial populations to global change perturbations [10], [66]. Our results show that overall the activity of phenol-oxidase – an enzyme responsible for breakdown of the structural components of plant material [67] – in litter was greater at sites receiving higher inputs of reduced N. This relationship was seen in both upland and lowland systems, the latter, however, stronger in areas of greater rainfall. Significant interactions between oxidised N and rainfall in upland systems were also apparent, possibly reflecting the wider span of oxidised N inputs (3–15 kg ha^−1^ yr^−1^) compared to lowland sites (3–9 kg ha^−1^ yr^−1^). The pattern of increased litter/soil enzyme activity with increasing N deposition supports results from a recent survey of biochemical indicators in lowland heathlands [19]. However, contrasting results have been reported previously for moorlands [18] and temperate forests [66], [67].

The positive relationship between N deposition – particularly reduced N - and litter PME activity provides the first field scale evidence of an increase in P demand across both upland and lowland heathland sites receiving high rates of N deposition. These findings are in line with those from manipulation experiments across a range of ecosystems [68], [69], and with survey evidence of increased plant uptake of P across transects of increasing N deposition in lowland heathland [15], [70].

The main factors regulating decomposition are moisture and temperature [6], [71]; this is also apparent in our survey results with enzyme activities highest in warmer, wetter areas. Climate change predictions for warmer, drier summers and cooler, wetter winters will, therefore, have implications for the activities of enzymes involved in the decomposition process, with likely consequences for nutrient availabilities and uptake. Concentrations of soil extractable NO_3_ and NH_4_ were significantly higher with increasing N deposition in the upland sites, although no such relationship was evident in the lowlands. The strong influence of climate on rates of nutrient turnover and availability [72], [73], and generally lower annual rainfall in lowland (600–1300 mm) compared to upland (800–2500 mm) sites, may explain the lack of relationship with N in lowlands and the generally higher abundance of litter in upland sites. This may be particularly the case in this survey since sampling was carried out in the summer when soil moisture levels are at their lowest and plant and microbial immobilisation of N are at their highest [74]. Caution must be exercised when making assumptions based on ‘snapshot’ measurements of mineral pools (particularly across broad temporal and spatial gradients) that are inherently subject to large inter-seasonal and inter-annual variations [75]. Furthermore, recent N-focused surveys carried out by Jones & Power [19] and Stevens *et al.* [76] found that soil type, underlying geology, climatic and altitudinal variation were important drivers of soil and plant responses, influencing the nature of relationships with N deposition.

Higher soil C:N ratios at sites with higher N inputs, specifically in the lowlands, indicate a shift towards immobilisation with increasing N deposition. Lower litter C:N ratios at high N sites contrast with this pattern, although may be explained by the previously mentioned N-driven stimulation at the early stage of decomposition where labile, energy rich substrates are abundant, followed by a slowdown in later stages as the relative proportions of recalcitrant compounds (such as lignin) progressively increase [77]. Whilst many studies of N deposition impacts have found that total soil N content increased with elevated N inputs [69], [78], [79], no evidence of N or C accumulation in relation to elevated N as a sole driver was found in this survey. This may reflect the depth (0–15cm) of the soil core sampled, which not only results in high levels of between site variability in depth of organic layer included in the sample (from <1cm to 15 cm), but may also be too deep to capture nutrient signals from recent decades that are present in the upper horizons.

Across all sites, the most significant driver influencing soil total N and C content was precipitation. Since climatic factors are known to strongly influence the production of plant material (and, subsequently, plant residues) [80], the dominant influence of rainfall is not surprising. Indeed, our results suggest that climatic variables are important drivers of many ecosystem processes and, therefore, are likely to influence responses to N deposition. Given that climate models predict altered precipitation and temperature patterns for the northern hemisphere [81], and that widespread exceedance of critical N loads is predicted across Europe for the foreseeable future [2], [82], the implications of global change for the biodiversity, structure and function of these internationally important heathland ecosystems are very considerable.

## Conclusion

The findings from this survey provide novel support to a growing body of evidence demonstrating a strong link between N deposition and an increase in nitrophilous plant species across a wide range of habitats [17], [22]. An associated decline in the diversity of both higher and lower plants across broad spatial and climatic gradients – in both upland and lowland environments - highlights the ubiquity of relationships with N, providing clear field evidence of widespread N-driven changes in the composition, structure and diversity of British heathlands. There is also strong evidence to suggest that current rates of N deposition are altering many biogeochemical processes, particularly rates of nutrient cycling and availability, and that these relationships are influenced by climatic conditions. Litter N concentrations, litter enzyme activity (phenol-oxidase and PME) and, for the uplands, soil extractable N concentrations, all appear to have consistent relationships with total N deposition and may, therefore, be useful biochemical indicators of, and responses to, N deposition. Given future predictions for climate change and continued wide scale exceedance of critical N loads across Europe, further effects of these global change phenomena on heathland diversity and associated ecosystem services can be expected.
